# Assessing the psychometric performance of the EQ-5D-5L among informal caregivers of people with dementia

**DOI:** 10.1007/s11136-024-03737-6

**Published:** 2024-07-24

**Authors:** Valeriia Sokolova, Jan Faller, Siti Khadijah Binti Mohamad Asfia, Lidia Engel

**Affiliations:** 1 School of Public Health and Preventive Medicine, Monash University Health Economics Group, Monash University, Melbourne, VIC Australia; 2 School of Health and Social Development, Deakin University, Burwood, VIC Australia

**Keywords:** Informal care, Caregiver burden, Dementia, Quality of life, EQ-5D-5L, Psychometric performance

## Abstract

**Purpose:**

This study aimed to examine the psychometric performance of the EQ-5D-5L in informal caregivers of people with dementia.

**Methods:**

Data were obtained from an online survey administered to informal caregivers of people with dementia in Australia. Known-group comparisons were examined by formulating 15 a priori hypotheses, where a difference was made between weak and strong hypotheses. Group comparisons were tested using the non-parametric Wilcoxon-rank and the Kruskal-Wallis test, as well as regression analysis. Floor and ceiling effects were considered to be present if more than 15% of respondents achieved the lowest or highest possible score, respectively.

**Results:**

In total, 212 informal caregivers of people with dementia were included in the analysis. On average, participants were 47 years old (SD: 17) and 61% of them were female. The mean EQ-5D-5L utility score was 0.88 (SD: 0.16) and the mean EQ-VAS was 72.47 (SD: 17.86). While there was no floor effect, 26% reported full health. Nine strong and three weak hypotheses were confirmed, supporting the ability of the EQ-5D-5L to discriminate between groups with respect to: self-reported health status, happiness levels, presence of mental or physical health conditions, ability to engage in enjoyable activities, and availability of support.

**Conclusion:**

Findings provide supporting evidence for the EQ-5D-5L in terms of its discriminant validity in informal caregivers of patients with dementia. However, the present ceiling effect suggests that the sensitivity of the EQ-5D-5L to detect improvements may be limited. Further studies are warranted examining other psychometric criteria, including reliability and responsiveness to change

## Background

Currently, it is estimated that there are over 55 million people worldwide living with dementia, with nearly 10 million new cases diagnosed every year [1]. Dementia is a neurodegenerative disease with various stages of severity characterized by symptoms of cognitive deterioration, tremors, movement problems and forgetfulness that appears to be one of the major causes of disability and dependency among older people globally [1]. Informal caregivers are usually children, partners or friends of people with dementia assisting them with oral communication, property maintenance, household chores to meal preparation, health and personal care [2]. Informal caregivers usually provide care without any payment, and they seldom receive enough training for these tasks as opposed to formal caregivers.

Caring for a person with dementia significantly impacts caregivers’ physical, mental health and social wellbeing, as people with dementia become increasingly unable to care for themselves and perform their usual activities [3]. In addition, caregivers of people with dementia often experience financial burden due to high health care expenses, reduced working hours, and psychosocial problems [4]. Compared to non-caregivers, caregivers of people with dementia report higher levels of stress, depression and anxiety symptoms [5, 6]. Various contributing factors, such as time spent caregiving [7, 8], severity of dementia [9] and increasing dependency of the person with dementia [9], further increase caregiver burden and lead to deterioration in caregiver’s health-related quality of life (HRQoL). However, more modelling studies are needed that control for, among other things, the age of the informal caregiver and disentangle decrement to HRQoL attributable to the time spent caring for a person with dementia and the decrement attributable to ageing alone [10].

The quality-adjusted life year (QALY) framework is commonly used in economic evaluations to capture the burden of disease. QALYs are estimated by multiplying the duration in a health state in years, by the health utility score for that health state. Health utility as a representation of HRQoL is often obtained through a generic preference-based measure. A preference-based measure consists of a classification system and a value set that is used to score responses to generate utility scores [11]. The classification system contains questions and response options, representing various HRQoL dimensions and corresponding severity levels. Responses to the classification system are used to assign people to a health state. A value set is then used to score the relative value of the health state to generate a utility value (index score), which is anchored on the 1-to-0 full health – dead scale, with values less than zero indicating that the health state is worse than being dead.

The EQ-5D is a generic preference-based measure of HRQoL typically used in economic evaluations [12]. The EQ-5D consists of five dimensions: mobility, self-care, usual activities, pain/discomfort, and anxiety/depression, where each dimension has three levels (EQ-5D-3L) or five levels (EQ-5D-5L) [12]. The EuroQoL Group developed the EQ-5D-5L as a means to improve the instrument’s ability to capture a more inclusive number of health states compared to the EQ-5D-3L [12]. The EQ-5D-5L instrument describes 3125 unique health states, which can be converted into utility values using established value set that exist for numerous countries [13]. These value sets were developed following the EuroQoL evaluation technique (EQ VT protocol) [14]. In addition to the five dimensions of the EQ-5D, which form the descriptive system, it also contains a visual analogue scale (EQ-VAS), which asks respondents to self-report their general health on a scale of zero (‘The worst health you can imagine’) to 100 (‘The best health you can imagine’) [13].

The EQ-5D has been both validated and widely used to measure spillover HRQoL of informal caregivers of care recipients with various diseases [15–20]. However, literature on informal caregivers of people with dementia is scarce [18, 21–23]. Although many economic evaluations in the area of dementia and Alzheimer’s disease include effects related to informal caregiving, preference is given to other generic and/or care-related QoL measures rather than EQ-5D [23]. Researchers tend to choose other measures due to concerns that the EQ-5D’s content might not fully reflect caregivers’ experiences [22]. Two studies [18, 21] concluded that the EQ-5D was a psychometrically robust instrument among informal caregivers of people with dementia, mild cognitive impairment in Parkinson’s disease and dementia with Lewy bodies, reporting good internal consistency and some level of convergent and construct validity. Contrary to this, Reed et al. [22] revealed that the EQ-5D-3L index score had a low sensitivity to change over an 18-month period and was not clearly differentiating by patient dementia severity. Generally, literature suggest that the EQ-5D may have limited sensitivity in measuring the impacts for dementia informal caregiver’s due to its focus on physical health [18, 21, 22].

Understanding the psychometric performance of the EQ-5D among informal caregivers is necessary to derive informal caregivers QALYs, which can be used to inform decision-making processes for interventions related to informal caregivers. Although health and social care interventions targeting patients can also have an effect on caregiver’s QoL, a systematic review of cost-effectiveness studies revealed that while caregivers’ time costs have been measured and valued in some studies, their health and well-being effects have been largely ignored in many economic evaluations [23]. As a result, the incremental cost-effectiveness ratio may be altered in either direction which may lead to different recommendations [23]. Given that the EQ-5D is the most commonly used measure and is often recommended by health technology assessment guidelines, it is important to explore its performance in informal caregivers. Additionally, the EQ-5D has been proposed by the task and finish group of the National Institute for Health and Care Excellence (NICE) in England to measure caregivers HRQoL in economic evaluations to ensure consistency with the methodology used to derive patient QALYs and to enable the aggregation of patient QALYs and caregiver QALYs to guide resource allocation decisions [24]. Thus, this study aimed to examine the psychometric performance of the EQ-5D-5L in informal caregivers of people with dementia.

## Methods

### Data source

Data were obtained from a previous study [25], which aimed to estimate the monetary value of informal care time provided to people with dementia using a discrete choice experiment (DCE) methodology. This Australia-wide study was conducted online and included both informal caregivers of people with dementia and members of the general public living in Australia. For the analyses reported in this paper, only data from informal caregivers were used. Informal caregivers were recruited between June – October 2021 via advertisements on websites by Dementia Australia, Carers Victoria, New South Wales, Queensland, Umbrella Dementia Cafés and Step up for Dementia as well as via the posts on their social media profiles (i.e., Twitter and Facebook). Additionally, a research market company Pureprofile was utilized to support recruitment. Informal caregivers were invited to complete an online survey, hosted by Pureprofile, which consisted of six parts: (i) screening questions and informal care characteristics; (ii) introduction to DCE attributes and levels; (iii) DCE choice tasks; (iv) demographic characteristics; (v) DCE debriefing and attribute ranking questions; (vi) questions on caregivers’ health and wellbeing, including the EQ-5D-5L. Ethics approval was granted by the *‘removed for peer review’* Human Research Ethics Committee (#2019-067), and all study participants had to agree that they have read the Information sheet and understand the purpose of the study before they could proceed with the survey.

### Statistical analyses

The psychometric criteria explored in this study were driven by data collected in the survey, and included exploration of floor and ceiling effects as well as known-group validity. Floor and ceiling effects refer to the number of respondents who achieved the lowest or highest possible score [26]. Floor or ceiling effects were considered to be present if more than 15% of respondents reported the lowest (55555) or highest possible (11111) health, respectively. Known-group validity is the extent to which the questionnaire can demonstrate different scores between known groups in a manner that is consistent with theoretically derived hypotheses [27]. According to Consensus-based Standards for the selection of health Measurement Instruments (COSMIN) guidelines [26] known-group validity was established when 75% of hypotheses were met.

A priori hypotheses about known groups were developed based on existing literature, previous research and discussion with the research team, which are detailed in Table 1. Hypotheses were categorized as weak and strong. Weak hypotheses were supported by inconsistent evidence or little evidence, whereas strong hypotheses were determined by consistent evidence-base. Known-group comparisons were made based on participant’s age, stage of dementia of care-recipient, employment, income level, number of weekly caregiving hours and current medical conditions. Further information about the measurement of these constructs and the corresponding survey questions are provided in Table 1. To test these hypotheses, we used the non-parametric Wilcoxon-rank test (for two-group comparison) and the Kruskal-Wallis equality-of-populations rank test (for three- or more group comparison), given that the EQ-5D-5L scores were not normally distributed but skewed to the right. All statistical analyses were performed in STATA version 17. The significance level was set as α = 0.05.

**Table 1:** A priori defined hypotheses for the assessment of known-group validity categorized according to strength of hypotheses (weak and strong)

Known-group comparison	Details and underlying evidence	Question in the survey
**Strong hypotheses**		
Self-rated health	Good self-reported health leads to an improvement in HRQoL [30, 39, 51].	In general, would you say your health is: • Excellent • Very good • Good • Fair • Poor
Happiness	Higher levels of happiness are associated with better caregiver’s HRQoL [30; 52].	How happy do you feel at the moment? Please place a mark on the scale below that indicates how happy you feel at the moment (0–10 scale).
Health condition	Caregivers’ underlying diseases and health conditions will negatively affect caregivers’ HRQoL [53–55].	Do you have any of the following ongoing medical conditions? (choose as many as apply) • No ongoing medical conditions • Anxiety • Arthritis • Asthma • Cancer • Chronic obstructive pulmonary disease (COPD) e.g. chronic bronchitis or emphysema • Chronic pain • Depression • Diabetes • Heart disease • High blood pressure or hypertension • Other (please specify)
Caregiving situation: Emotional and physical exhaustion	Higher levels of emotional and physical exhaustion are associated with lower HRQoL [56].	I feel emotionally and physically exhausted in my caring role: • None of the time • Sometimes • Most of the time
Caregiving situation: Relational problems	Relationship problems based on an caregiver’s perception are associated with lower caregiver HRQoL [57].	I have relational problems with the person receiving care: • None of the time • Sometimes • Most of the time
Caregiving situation: Difficulty getting support	Lack of support with the caregiving role is associated with lower caregiver HRQoL [33, 53].	I have difficulties getting social and practical support with my caring role: • None of the time • Sometimes • Most of the time
Caregiving situation: Enjoyable activities	There is a positive association between caregivers’ ability to perform enjoyable activities, their independence and HRQoL [35, 58, 59].	I am unable to do the things that I enjoy in life due to my caring role: • None of the time • Sometimes • Most of the time
Age	Older age is associated with poorer HRQoL [18, 40, 45].	What is your current age (in years)?
Stage of dementia	Greater dementia severity suggests greater level of dependency and hence lower caregiver HRQoL [22].	To the best of your ability, how would you rate this person’s level of dementia? • Mild or early-dementia • Moderate or middle-stage dementia • Severe or late-stage dementia
Number of hours in caregiving	The longer number of hours dedicated to caregiving, the lower caregiver’s HRQoL [22; 38].	In total, how many hours did you spend on helping or supporting this person (e.g. personal care, shopping or emotional support) in the last 7 days? • Less than 10 h per week • 10–19 h per week • 20–29 h per week • 30–39 h per week • 40 h per week or more
***Weak hypotheses***		
Length of caregiving	The longer period of caregiving, the lower caregiver’s HRQoL [36, 37].	How long have you been providing care or support to this person? • Less than 1 month • 1–6 months • 7–12 months • 13–24 months • More than 2 years
Income level	The lower the income, the greater the impact of financial barriers and lower HRQoL [60].	What is your best estimate of the total household income received by all household members, from all sources, before taxes and deductions, in the past 12 months? • Negative or zero Income • $1 - $29,999 per year • $30,000 - $59,999 per year • $60,000 - $89,999 per year • $90,000 - $119,999 per year • $120,000 - $149,999 per year • $150,000 or more per year • Prefer not to answer • Don’t know
Employment	Caregivers who maintain employment alongside their caregiving role experience better HRQoL, where employment is hypothesized to be protective against the responsibilities of care and caregiving stress [61].	What is your employment status? • Employed (full-time or part-time) • Unemployed • Student (full-time or part-time) • Retired • Housewife/husband • Other • Prefer not to answer
Relationship with person with dementia	Child and spousal caregivers experience higher caregiver burden and poorer HRQoL compared to friends and distant relatives [8, 45, 49].	What is your relationship to this person? She/he is: • My partner • My father or mother • My son or daughter • My grandparent • Another family member • My neighbour • My friend • Other
Number of people in household (below age 16)	Taking care of other household members (below age of 16) can add additional family responsibilities and increase caregiver burden and stress, which results in lower HRQoL [31, 53].	How many people under 16 years live in your household?

## Results

### Sample characteristics

The characteristics of participants are presented in Table 2. Of 212 informal caregivers, 61% were female. On average, the participating caregivers were 47.4 (SD: 17.0) years old and were mostly children (37.3%) or spouses (21.7%) of the person with dementia. About 52% held a university degree. More than half (59.2%) of participants were still full-time or part-time employed. The majority of them (68.8%) had at least one medical condition. Almost half (44.3%) of individuals reported that they have been providing care for more than two years; the majority spent less than 10 h (30.2%) or 10–19 h (28.3%) per week on caregiving tasks. Additional caregiving characteristics are detailed in Table 3.

**Table 2:** Descriptive statistics of study participants

Characteristics	Caregiver of person with dementia (*n* = 212)
**Female, n (%)**	130 (61.3)
**Age, mean (SD), y** **Age, median (IQR), y**	47.4 (17.0) 46 (33-61.5)
**Marital status N (%)**	
Single	50 (23.6)
Married	131 (61.8)
De facto	14 (6.6)
Divorced	11 (5.19)
Separated	2 (0.94)
Widowed	2 (0.94)
Other	22 (0.94)
**Employment N (%)**	
Employed (full-time or part-time)	116 (59.2)
Unemployed	80 (40.8)
**Education level N (%)**	
Year 10 or less	18 (8.5)
Year 11/12	24 (11.3)
Cert III/IV or Diploma	43 (20.3)
University degree	111 (52.4)
Other	16 (7.5)
**Income level N (%)**	
$0-$59,999	80 (42.3)
$60,000-$119,999	56 (29.6)
$120,000≥	53 (28.0)
**Country of birth**	
Australia	163 (76.9)

**Table 3:** Caregiving characteristics of study participants

Characteristics	Caregiver of person with dementia (*n* = 212)
**Relationship with person with dementia ** *N* ** (%)**	
Partner	46 (21.7)
Parent	79 (37.3)
Child	1 (0.47)
Grandparent	35 (16.5)
Relative	24 (11.3)
Neighbor	9 (4.3)
Friend	13 (6.1)
Other	5 (2.4)
**Household members under 16 years old N (%)**	
Yes	83 (39.2)
No	129 (60.9)
**Caring hours per week N (%)**	
Less than 10 h per week	64 (30.2)
10–20 h per week	60 (28.3)
20 h per week or more	88 (41.5)
**Length of caregiving N (%)**	
Less than 12 months	83 (39.2)
More than 12 months	129 (60.9)
**Person’s level of dementia severity N (%)**	
Mild or early-dementia	97 (45.75)
Moderate or middle-stage dementia	89 (41.98)
Severe or late-stage dementia	26 (12.26)
**Existing medical condition(s) N (%)**	
None	66 (31.1)
One	59 (27.8)
More than one	87 (41.0)

Other refers to unknown subgroups

The mean EQ-5D-5L health utility score was 0.88 (SD: 0.16). The maximum utility score was 1.00 and the minimum − 0.124. The mean EQ-VAS score 72.47 (SD: 17.86), the median EQ-VAS reached 75.0 (interquartile range (IQR) = 26.5), with scores ranging from 20 to 100.

**Fig. 1:**
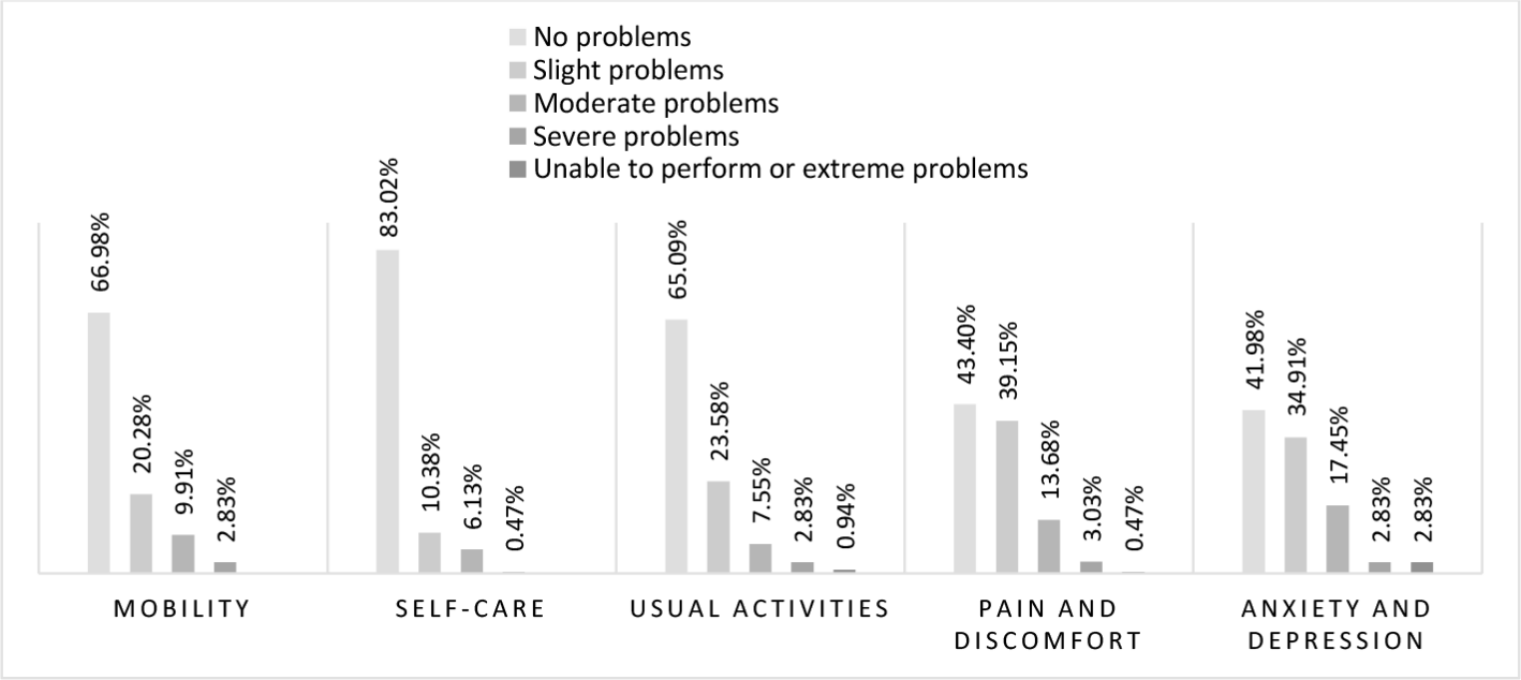
The percentage of participant responses to each domain

The distribution of participant responses to each dimension of the EQ-5D-5L is shown in Fig. 1, where 4% of informal caregivers reported extreme problems in at least one dimension, while there were no individuals with extreme problems in more than two dimensions at the same time. Extreme problems were reported for anxiety/depression (3%), usual activities (1%), pain/discomfort (0.5%). The highest number of respondents reporting ‘no problems’ was obtained for the self-care dimension (83%), followed by mobility (67%), usually activities (65%), pain/discomfort (43%), and anxiety/depression (42%).

### Floor and ceiling effects

There were no floor effects present as none of participants selected the lowest (floor) possible scores (i.e., health profile 55555) across all EQ-5D-5L dimensions. However, there was an indication of substantial ceiling effects, where 54 individuals (25.5%) reported full health (i.e., health profile 11111). The most commonly reported EQ-5D-5L health profiles are shown in Table 4.

**Table 4:** Frequency of the most reported EQ-5D-5L profiles

Combination method	Number of cases	Percentage (%)	Cumulative percentage (%)
11111	54	25.47	25.47
11112	22	10.38	35.85
11122	17	8.02	43.87
11121	10	4.72	48.58
11123	4	1.89	50.47
…	…	…	…
44212	1	0.47	100.00
Total	**212**	**100.00**	

### Known-group validity

Out of the ten strong hypotheses, nine were confirmed (see Table 5). There were statistically significant differences in EQ-5D-5L utility scores between groups with respect to: age (*p* < 0.05), self-reported health status (*p* < 0.001), happiness levels (*p* < 0.001), presence of mental and/or physical health conditions (*p* < 0.001), hours of care per week (*p* < 0.01) and various caregiving situations (emotionally and physically exhausted, *p* < 0.001; relationship problems, *p* < 0.01; difficulties getting social support, *p* < 0.001; and unable to do things they enjoy, *p* < 0.05). Caregivers with excellent self-reported health tended to have higher EQ-5D-5L scores compared with those reporting good, fair or poor health. Self-reported happiness was positively associated with higher HRQoL. On the contrary, informal caregivers with an existing mental and/or physical health condition reported lower EQ-5D-5L scores compared to those without. Emotional and physical exhaustion, relationship problems with the care recipient, inability to get social support, and inability to do enjoyable activities were negatively associated with caregivers EQ-5D-5L scores. The more hours of care informal caregivers provided per week, the lower were their EQ-5D-5L scores. While the EQ-5D-5L was able to discriminate between caregivers of people with mild dementia compared with moderate/severe dementia, it was unable to discriminate between moderate and severe dementia.

**Table 5:** Known group comparisons for caregivers of dementia – strong hypotheses

Characteristics	*N*	ED-5D-5L		
		Mean	Standard deviation	*p*-value
**Self-rated health**				
Excellent	33	0.92	0.13	< 0.0001
Very good	73	0.92	0.10	
Good	67	0.89	0.14	
Fair	31	0.80	0.21	
Poor	8	0.59	0.36	
**Health condition**				
None	66	0.97	0.06	< 0.0001
One	59	0.89	0.14	
More than one	87	0.81	0.19	
**Caregiving situation: Emotionally and physically exhausted**				
None of the time	22	0.91	0.14	0.0006
Sometimes	126	0.91	0.13	
Most of the time	64	0.82	0.22	
**Caregiving situation: Relationship problems**				
None of the time	70	0.92	0.12	0.0013
Sometimes	103	0.88	0.16	
Most of the time	39	0.80	0.23	
**Caregiving situation: Difficulties getting social support**				
None of the time	69	0.93	0.10	0.0009
Sometimes	106	0.87	0.16	
Most of the time	37	0.80	0.23	
**Caregiving situation: Unable to do things you enjoy**				
None of the time	47	0.93	0.09	0.0267
Sometimes	114	0.88	0.15	
Most of the time	51	0.83	0.23	
**Stage of dementia**				
Mild or early-dementia	97	0.90	0.15	0.0999
Moderate or middle-stage dementia	89	0.86	0.18	
Severe or late-stage dementia	26	0.86	0.15	
**Caring hours per week**				
Less than 10 h per week	64	0.92	0.13	0.0055
10–20 h per week	60	0.91	0.10	
20 h per week or more	88	0.83	0.20	
**Age**		**β**	**SE**	**p-value**
	Constant	0.94	0.03	0.041
	Age	-0.001	0.001	
	Adjusted R^2^	0.015		
**Happiness**				
	Constant	0.65	0.04	< 0.0001
	Happiness	0.03	0.005	
	Adjusted R^2^	0.15		

Three out of five (60%) weak hypotheses were confirmed (see Table 6). The longer informal caregivers provided support for the person with dementia, the lower were their EQ-5D-5L scores (*p* = 0.007). Friends and distant relatives experienced less caregiver burden, hence had higher EQ-5D-5L scores compared to spousal and child caregivers (*p* < 0.001). Full-time or part-time employment was associated with higher EQ-5D-5L scores of informal caregivers compared with unemployment (*p* = 0.007). However, no difference was found by income level and number of household members under 16 years old.

**Table 6:** Known group comparisons for caregivers of dementia – weak hypotheses

Characteristics	Count	ED-5D-5L		
		Mean	Standard deviation	*p*-value
**Length of caregiving**				
Less than 12 months	83	0.92	0.11	0.007
More than 12 months	129	0.86	0.19	
**Income level**				
$0-$59,999	80	0.86	0.17	0.0544
$60,000-$119,999	56	0.88	0.19	
$120,000≥	53	0.90	0.15	
**Employment**				
Employed (full-time or part-time)	116	0.90	0.13	0.0067
Unemployed	80	0.85	0.18	
**Relationship with person with dementia**				
Partner/parent/child	126	0.86	0.17	< 0.0001
Friend/other	86	0.91	0.16	
**Household members under 16 years old**				
No	129	0.87	0.18	0.0545
Yes	83	0.90	0.13	

## Discussion

This study examined the psychometric performance of the EQ-5D-5L in informal caregivers of people with dementia in Australia. Overall, 12 out of 15 (80%) a priori hypotheses were confirmed. There was strong evidence that the EQ-5D-5L was able to discriminate between groups of informal caregivers based on known-group validity. However, the present ceiling effect implies that the EQ-5D-5L might have limited sensitivity to detect improvements among caregivers. The mean EQ-5D-5L score (0.88) in our study population was also comparable to the population norms in Australia (0.86) [28] indicating that it may be less sensitive in capturing the full burden that caregivers experience.

Informal caregivers of people with dementia have to deal with some unique challenges due to the prolonged course of the disease, progression of person’s disability, and lack of a cure [29]. In our analysis, caregivers reporting higher happiness scores and caregivers with good self-reported health had higher EQ-5D-5L scores, emphasizing the importance of prioritizing the physical and mental health of caregivers. Subjective happiness has been known to be remarkably related to depression, which is a predictive factor of HRQoL among family caregivers [30]. Since depression (and anxiety) is one of the 5 components of EQ-5D, it is not surprising that the improvement in the score for the depression component of the EQ-5D leads to improved HRQoL scores. Engaging in self-care and the ability to cope are essential in maintaining good health and wellbeing of informal caregivers while continuing to provide care [29, 31]. Informal caregivers with existing medical conditions reported lower EQ-5D-5L scores, which potentially hinder them from providing thorough care for the person with dementia resulting in higher levels of stress, anxiety and lower HRQoL. Previous literature showed that the time-consuming nature of caring and lack of time to seek professional support limit the capacity of caregivers to maintain their mental and physical health [31]. Individuals with preexisting chronic illnesses reported that their condition had worsened after becoming an informal caregiver [31], emphasizing the burden of caring.

Our study also found that those having relational problems with the person with dementia reported lower EQ-5D-5L scores. Transition to a caregiver role, shift in responsibility, behavioral and communication problems of the person with dementia, especially as the disease progresses, affect the existing relationship [29], which can negatively impact carer’s HRQoL and hence EQ-5D-5L scores. Often informal caregivers might be reluctant to share some of the relational changes they are experiencing, leaving them feeling lonely and unsupported [32]. Our results confirmed that caregivers reporting difficulties in getting social support lead to lower EQ-5D-5L scores in informal caregivers of people with dementia. While the caregiving role often limits time for social interaction and building new relationships, the ability to access social support may be a beneficial coping strategy for informal caregivers [29, 33]. Sufficient social support can facilitate caregivers to dedicate more time towards resting, socializing and engaging in leisurely activities, which could improve their HRQoL and make their caring responsibilities more manageable [31]. Furthermore, our study revealed that inability to perform enjoyable activities results in lower EQ-5D-5L scores in informal caregivers. Providing care for a person with dementia often limits caregiver’s time for their own hobbies and interests, further restricting opportunities to relax or to release built-up pressure [29]. Many informal caregivers report loss of freedom and self-identity beyond caring [31]. Encouraging caregiver’s independence and participation in activities providing pleasure and a sense of accomplishment while maintaining social relationship is a crucial part of caregiver’s support [34].

While we expected that EQ-5D-5L scores would decrease with greater level of dementia severity, the severity of dementia did not appear to impact caregiver’s HRQoL, although this tendency has previously been established in the literature [22]. It is not inconceivable that the greatest decrease in informal caregiver’s HRQoL may be observed in those with moderate dementia because people often develop more behavioral problems during this stage of the disease [22, 29, 35]. By the time the individual has progressed to more severe dementia, caregivers most likely have adapted their lifestyle and/or transitioned to formal care. This could possibly also explain why in our study the EQ-5D-5L was able to discriminate between caregivers of people with mild dementia compared with moderate/severe dementia but not between moderate and severe dementia.

In terms of duration of care, as hypothesized, longer duration of caregiving was associated with the lower EQ-5D-5L scores informal caregiver’s probably because cognitive status of a person with dementia worsens and care demands increase over time [36, 37]. Typically, as dementia progresses, informal caregivers not only help with the daily living activities (e.g., household, financial and social activities) but also with personal care, and provide constant supervision [29]. Duration of care may also be associated with lower HRQoL in caregivers because it may reflect the accumulated toll of providing care over many years for the person with dementia [36]. An empirical analysis of cross-sectional data from 8 European counties revealed that there was a significant positive relationship between caregiving hours and decreased psychological wellbeing in informal caregivers of people with dementia in Estonia, Finland, France, Sweden and the United Kingdom [38]. Dedicating longer hours on weekly caregiving may result in individuals facing lack of social interaction, personal freedom and independence, which can lead to lower HRQoL and hence, EQ-5D-5L scores. However, more modelling studies are needed that control for, among other things, the age of the informal caregiver and disentangle decrement to HRQoL attributable to the time spent caring for a person with dementia and the decrement attributable to ageing alone.

The current study demonstrated that caregiver’s increased age was associated with lower EQ-5D-5L scores, although the effect size was low. These findings were similar to previous studies conducted by Farina et al. [39]; Contreras, Mioshi, and Kishita [40], which concluded that the associations between caregiver age and HRQoL were unclear and required further exploration.

Although in this caregiver sample, friends and distant relatives reported better EQ-5D-5L scores, previous studies suggest that closeness of dyadic relationship could predict both beneficial [31, 41–43] and adverse [31, 41, 44, 45] caregiver outcomes. Adverse effects of having a closer relationship with the person with dementia are higher levels of emotional involvement and psychosocial stress, which negatively impact caregiver’s physical health and psychological well-being [32]. Couples and relatives may experience a significant shift in family dynamics, including increasing distance, reduced communication, and lack of intimacy and affection [31, 32]. Also, close relatives and spouse caregivers more often experience shared financial concerns which could contribute to the caregiver burden [45]. In later stages of dementia, strengthening family and/or friend relationships may provide an additional support for the informal caregivers as the reciprocity in the dyad relationship is challenged [32]. On the other hand, loss of closeness over the course of caregiving may help caregivers to develop an adaptive mechanism over time and protect their well-being from deterioration. Some informal caregivers may perceive caring as a source of personal development and inner strength, which might contribute to improved HRQoL [31].

Full-time or part-time employment was associated with higher EQ-5D-5L scores, potentially due to the sustained sense of independence and increased opportunities for social activities associated with going to work. Nevertheless, the relationship between caregiver’s income and their HRQoL might need to be explored further as its effects are probably confounded by additional sources of income, caregiver’s support, and government funding.

Also, taking care of other household members (below age of 16) on top of caring for the person with dementia has not been associated with lower caregiver’s EQ-5D-5L scores. While this could add additional family responsibilities and increase caregiver burden and stress, if those family members are independent enough, they could provide additional emotional support and help with the caregiving duties.

The obtained results are in line with the evidence from the literature on EQ-5D psychometric properties in dementia caregivers [18, 21, 22]. Vatter et al. [21] revealed that EQ-5D-3L was a robust instrument showing good internal consistency and convergent validity, despite possessing a ceiling effect. However, Reed et al. [22] expressed a concern that EQ-5D may not be the optimum measure of the impact of caring for people with Alzheimer’s dementia due to its focus on physical health. Mental and social wellbeing are a big part of caregiver burden, and the EQ-5D-5L might not capture such health concerns experienced by dementia informal caregivers like emotional stress and loneliness.

Even though this study did not include a comparison with other QoL measures, existing literature suggests that in general the EQ-5D-5L is a relatively sensitive outcome measure for informal caregivers [15, 17, 18]. Bhadhuri et al. [15] demonstrated that the EQ-5D-5L had greater construct validity and responsiveness by detecting stronger associations than the SF-6D for spillovers in informal caregivers of patients with meningitis. In addition, EQ-5D-5L had satisfactory reliability and validity in family caregivers of leukemia patients [17]. Further, McLoughlin et al. [18] and McCaffrey et al. [46] tentatively concluded that care-related QoL measures (the Carer Experience Scale (CES), CarerQoL-7D, and ASCOT-Carer) were better than the EQ-5D-5L in detecting impacts of caring on HRQoL. The CES measures caregiver’s experience more broadly, the CarerQol focuses on caregiver burden, while the ASCOT-Carer captures aspects of carers’ QoL that may be influenced by social care services and support. Each of the instruments demonstrated good discriminant validity, and some degree of reliability and feasibility [46]. The ASCOT-Carer exhibited good internal consistency and test-retest reliability [46]. Hence, the idea of focusing on outcomes broader than health for caregivers might be more appropriate. Future direct (head-to-head) comparisons between the EQ-5D-5L and care-related QoL measures are warranted.

## Limitations

As this study was a secondary data analysis, not all psychometric criteria could be examined due to data unavailability. These include: reliability, convergent validity and responsiveness to change. Additionally, since all data were derived from the online survey that included forced choices, we were unable to explore missing values and hence examination of the practically of the EQ-5D-5L. As with all online surveys, a further limitation includes the absence of confirming the caregiver status of respondents completing the survey. Since the survey was self-reported by caregivers we had no further information about the disease severity of the person with dementia. Characteristics like personality traits were not assessed for differences between respondents. Hunt et al. [47] revealed that neuroticism was negatively associated with how caregivers rate their capability to ‘live well’ while conscientiousness and extraversion had a positive impact on caregivers’ HRQoL scores.

There is also a concern regarding generalizability of the results due to unequal distribution of age, gender and other characteristics of our sample, where recruitment was based on a convenience sample rather than a representative sample. Therefore, the obtained results have to be interpreted with caution.

The analysis was based on 212 participants, which is a relatively small sample size. However, according to Frost, Reeve, Liepa, Stauffer, and Hays [48] the sample size of at least 200 people is sufficient for basic psychometric analysis, and previous EQ-5D-5L evaluations in dementia caregivers included even less participants [18; 21].

Future studies should examine other psychometric properties of the EQ-5D-5L among informal caregivers of patients with dementia, such as reliability and responsiveness to change. Additionally, future head-to-head comparisons are warranted with other generic measures (e.g., Short-Form 6-Dimension (SF-6D)) or informal caregiver specific measures, such as the C-DEMQOL [49] and Scales measuring the Impact of DEmentia on CARers (SIDECAR) [50] developed for caregivers of people with dementia. A more thorough investigation would further increase knowledge about the accuracy and appropriateness of EQ-5D-5L questionnaire in this population. Moreover, additional caregiver data are required to better reflect the dynamic of spillover effects in relation to disease severity, end-of-life care, and care setting.

## Conclusion

In summary, this study provides supporting initial evidence for the EQ-5D-5L in terms of its discriminant validity in informal caregivers of people with dementia in Australia. However, the present ceiling effect suggests that the sensitivity of the EQ-5D-5L to detect improvements among caregivers may be limited. Further studies are warranted to examine other psychometric criteria, including reliability and responsiveness to change.
